# Retro-2 alters Golgi structure

**DOI:** 10.1038/s41598-022-19415-x

**Published:** 2022-09-02

**Authors:** Xihua Yue, Bopil Gim, Lianhui Zhu, Chuanting Tan, Yi Qian, Morven Graham, Xinran Liu, Intaek Lee

**Affiliations:** 1 School of Life Science and Technology, ShanghaiTech University, Shanghai, China; 2 School of Physical Science and Technology, ShanghaiTech University, Shanghai, China; 3 University of Chinese Academy of Sciences, Beijing, China; 4 Department of Cell Biology, Yale University School of Medicine, New Haven, CT 06520 USA

**Keywords:** Cell biology, Membrane trafficking, Organelles

## Abstract

Retro-2 directly interacts with an ER exit site protein, Sec16A, inhibiting ER exit of a Golgi tSNARE, Syntaxin5, which results in rapid re-distribution of Syntaxin5 to the ER. Recently, it was shown that SARS-CoV-2 infection disrupts the Golgi apparatus within 6–12 h, while its replication was effectively inhibited by Retro-2 in cultured human lung cells. Yet, exactly how Retro-2 may influence ultrastructure of the Golgi apparatus have not been thoroughly investigated. In this study, we characterized the effect of Retro-2 treatment on ultrastructure of the Golgi apparatus using electron microscopy and EM tomography. Our initial results on protein secretion showed that Retro-2 treatment does not significantly influence secretion of either small or large cargos. Ultra-structural study of the Golgi, however, revealed rapid accumulation of COPI-like vesicular profiles in the perinuclear area and a partial disassembly of the Golgi stack under electron microscope within 3–5 h, suggesting altered Golgi organization in these cells. Retro-2 treatment in cells depleted of GRASP65/55, the two well-known Golgi structural proteins, induced complete and rapid disassembly of the Golgi into individual cisterna. Taken together, these results suggest that Retro-2 profoundly alters Golgi structure to a much greater extent than previously anticipated.

## Introduction

The Golgi apparatus plays important roles in post-translational modifications of newly synthesized proteins and is also considered as a major sorting center at the cross-road of the secretory and the endocytic pathways^1,2^. Its stacked architecture, a hallmark feature of the Golgi apparatus, is crucial for its post-translational role and is known to be mediated by a group of Golgin tethers, including GM130 and Golgin45, and two Golgi Reassembly and Stacking proteins (GRASPs; GRASP65/55), and the Golgi undergoes dynamic disassembly and reassembly during mitotic cell division^3–9^.

GRASPs-mediated Golgi stacking by their PDZ domain interaction in trans had been widely accepted in the field^10,11^. However, two recent studies conclusively showed that GRASPs are not required for Golgi stacking in vivo, but necessary for Golgi ribbon formation^12–14^. On the other hand, Golgins, such as GM130, seem to be essential for SNARE-mediated membrane fusion of mitotic Golgi membranes during post-mitotic Golgi reassembly^15–17^. GM130 had been shown to directly interact with two Rab-GTPases, Rab1 and Rab33b as well as Golgi *t*SNARE Syntaxin5^3,8,15,18^. While it is still largely unknown by what mechanism these Golgins contribute to Golgi stack assembly or maintenance, recent studies suggest that many of these Golgin tethers may phase-separate to form liquid-like condensates, potentially contributing to Golgi stack assembly/organization^19,20^. Overall, exact mechanism of cisternal stacking/adhesion still remains elusive after many years of research.

We had previously shown that the two Syntaxin5-interacting Golgins, GM130 and Golgin45, could substitute for GRASPs to such an extent that their exogenous over-expression could create morphologically and functionally normal Golgi stacks in GRASP65/55-depleted mammalian cells, suggesting that the two Golgins and GRASPs may play extensively complementary roles in Golgi structure maintenance^21^. Under steady state condition, the more enigmatic Golgin, Golgin45, forms a multimeric protein complex with Acyl-CoA Binding-Domain-3 (ACBD3), GRASP55, Rab2-GTP and TBC1D22, a Rab-GTPase activating protein, which seems to assist in domain organization and regulate Golgi structural proteins^22^.

In addition, Golgin45 was also shown to recruit Tankyrase1 (TNKS1), a regulator of Wnt-signaling and telomere maintenance, to the Golgi and is subjected to TNKS1-dependent poly (ADP-Ribosyl)ation and subsequent proteasomal degradation, which greatly influences anterograde cargo secretion and protein N-glycosylation^23^. Strikingly, inter-cisternal membrane fusion frequently occurs in cells simultaneously depleted of GM130 and Golgin45 or in cells transfected with a Golgin45 mutant (D171A) that abrogates the interaction between Golgin45 and Syntaxin5, suggesting that Golgin-Syntaxin5 interaction may contribute to structural integrity of the Golgi stack during interphase^24^. In that study and a previous study by Wang’s group, RNAi-mediated depletion of Golgin45 and GRASP55 were shown to result in statistically significant reduction in the number of cisterna per stack^24,25^. Overall, these studies point to a possibility that Golgin45 and GRASP55 may not be required for stacking, but they may contribute to cisternal adhesion incrementally in an additive manner, as proposed previously^21^.

These results also raised further questions regarding whether Golgin-Syntaxin5 interaction may play more direct roles in Golgi structure maintenance by supporting cisternal adhesion. Since prolonged knockdown of Syntaxin5 by RNA interference for 24–48 h may be disruptive to entire secretory pathway, a direct knockdown approach was not a viable option to investigate this hypothesis.

A small molecule drug-like inhibitor, “Retro-2” has recently been shown to acutely re-distribute Syntaxin5 from the Golgi to the ER within 3–4 h by blocking Syntaxin5 interaction with an ER exit site protein, Sec16A^26^. We hypothesized that this ability of Retro-2 to rapidly displace Syntaxin5 from the Golgi could be exploited as a type of ‘knock-sideway’ approach, which may allow us to investigate the structural role of Syntaxin5 without actually knocking down this important Golgi *t*SNARE.

Thus, in this study, we used Retro-2 and electron microscopy to re-examine the structural role of Syntaxin5 in Golgi stack maintenance. Our new results show that Retro-2 treatment leads to partial disassembly of the Golgi stack without influencing protein secretion of both small and large cargos. Importantly, Retro-2 induces rapid disassembly of the Golgi stacks in GRASP65/55-depleted HeLa cells, resulting in accumulation of COPI-like vesicular profiles and unstacked Golgi cisterna, whose morphologies were highly similar to rapidly unstacked Golgi membranes in SARS-CoV-2 infected cells^27^. Taken together, these results suggest that Syntaxin5 may assist in Golgi cisternal adhesion and maintenance of Golgi structure during interphase by interacting with various Syntaxin5-binding Golgin tethers.

## Results and discussion

### Retro-2 treatment results in accumulation of COPI-like vesicles at the Golgi

**Figure 1:**
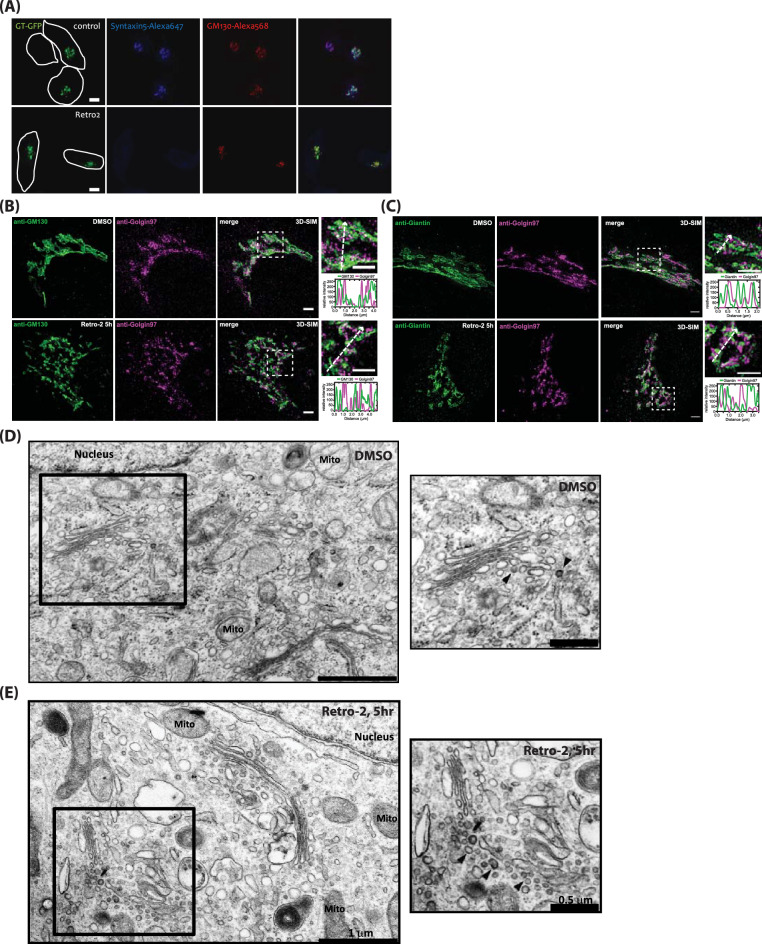
Acute displacement of Syntaxin5 by Retro-2 results in significant turnover of Golgi membranes to vesicular profiles, accumulated in peri-Golgi area. (**A**) Retro-2 treatment re-distributes Syntaxin5 from the Golgi within ~ 5 h. Confocal micrographs showing that Syntaxin5 is largely absent at the Golgi in HeLa cells treated with 25 μM Retro2 for 5 h. Golgi markers, GT-GFP and GM130, did not show any noticeable change in Retro2-treated cells. (scale bar 10 μm). (**B**,**C**) 3D-SIM images of the Golgi, stained with anti-GM130 (*cis*-Golgi) and anti-Golgin97 (*trans*-Golgi) or anti-Giantin (Golgi rim) and anti-Golgin97 antibodies in cells treated with either DMSO (control) or 25 μM Retro-2 for 5 h. Note that Golgi ribbon structure still localized to perinuclear region in both DMSO and Retro-2 treated cells (scale bar 2 μm). (**D**,**E**) Representative EM photos showing accumulation of vesicular profiles in Retro-2-treated cells. We observed striking amount of vesicular profiles in Retro-2-treated cells, but failed to notice any significant Golgi disassembly or cisternal dilation, as often observed in GRASP or Golgin knockdown experiments. *M* mitochondria, *N* nucleus, *G* Golgi apparatus (scale bar 1 μm).

In order to confirm rapid displacement of Syntaxin5 from the Golgi by Retro-2, we transfected HeLa cells with a Golgi resident protein, β(1, 4)-galactosyltransferase fused to GFP (GT-GFP), overnight, followed by treatment with either DMSO (control) or 25 μM Retro-2 for 5 h. Cells were then fixed and stained with anti-Syntaxin5 and anti-GM130 antibodies for confocal study. The results showed that Retro-2 treatment indeed resulted in almost complete displacement of Syntaxin5 from the Golgi after 5 h, while a Golgi glycosyltransferase, GT-GFP, and endogenous GM130 remained at the Golgi (Fig. 1A). There was no apparent morphological change of the Golgi under confocal microscope in Retro-2-treated cells, compared to DMSO control, despite of acute displacement of Syntaxin5 from the Golgi.

Due to insufficient resolution of conventional confocal microscopy, we opted to use structured illumination microscopy (SIM), a type of super-resolution microscopy, to further characterize the effect of Syntaxin5 displacement from the Golgi complex by Retro-2. Thus, we treated HeLa cells with either DMSO (control) or Retro-2 for 5 h, followed by fixing and staining with anti-GM130 (a *cis*-Golgi marker) or anti-Giantin antibody (a Golgi rim marker) and anti-Golgin97 antibody (a *trans*-Golgi marker) to examine *cis–trans* organization (GM130/Golgin97 pair) and overall influence on Golgi architecture (Giantin/Golgin97 pair) by 3D-SIM. The results showed that Retro-2 treatment appeared to result in partial disruption of the Golgi ribbon structure, while perinuclear localization of the Golgi apparatus was largely unaffected, suggesting that Retro-2 treatment seems to disrupt the Golgi ribbon structure, but does not lead to significant disassembly of the Golgi stack or disruption of *cis–trans* organization (Fig. 1B,C).

As it was difficult to judge the precise nature of this structural change even via super-resolution microscopy, we prepared the cells for examination by electron microscopy. HeLa cells were treated either DMSO or Retro-2 for 5 h, followed by fixing and embedding for EM study. The results showed striking changes in Golgi structure and morphology. While the Golgi ribbon structure still appeared to be concentrated within the perinuclear region of the cells, Retro-2-treated cells displayed a large number of vesicular profiles accumulated near the Golgi (Fig. 1D,E; black arrowheads in insets). Note that these vesicular profiles were also observed in control cells, albeit at much less quantities, suggesting that a significant portion of Golgi membranes may have been turned over and replaced by these vesicles.

### COPI-like vesicular profiles accumulate in the peri-Golgi region of HeLa cells treated with Retro-2

**Figure 2:**
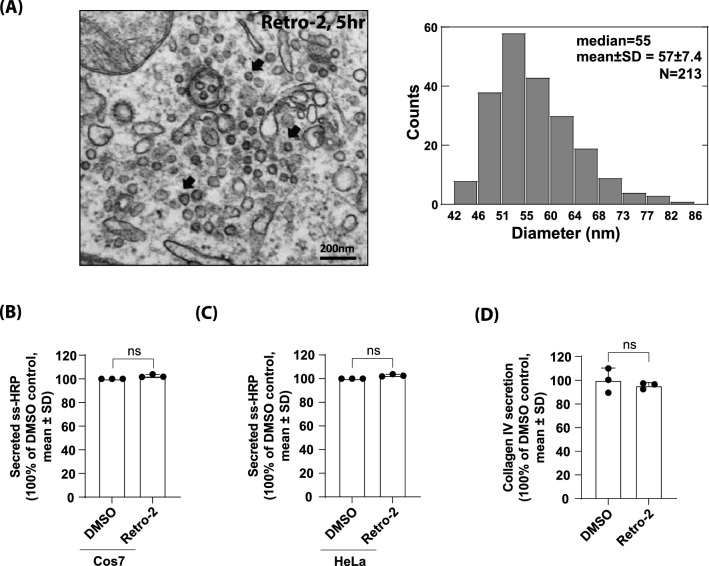
Retro-2 treatment induces COPI-like vesicles accumulation in the peri-Golgi region and does not affect anterograde secretion of cargo proteins. (**A**) Size distribution analysis of Retro-2-induced vesicular profiles suggest that a large fraction of these accumulated vesicles are likely to be uncoated COPI vesicles. For diameter distribution of the vesicles, we measured maximum diameter from membrane to membrane. Distribution median value is 55 and mean value is 57 nm (n = 213) (scale bar 200 nm). (**B**,**C**) Retro-2 treatment does not affect secretion of soluble secretory cargo, ss-HRP, in HeLa or COS7 cells. Cells were transfected with ss-HRP overnight, then the cells were changed with fresh medium and treated with DMSO or Retro-2 for 5 h. The activity of secreted HRP was estimated measured by TMB ELISA, as described in the methods. Relative secreted ss-HRP (mean ± SD) was shown and experiments were repeated for three times. Statistical analysis was performed using two-tailed, paired t.test (mean ± SD, *n.s.* not significant). (**D**) Retro-2 treatment does not affect secretion of large cargo, collagen IV, in COS7 cells. COS7 cells pre-treated with DMSO or Retro-2 for 2 h before folding block (40 °C, 3 h) without ascorbate and transport pulse condition, was later induced by shifting cells to 32 °C in the presence of 100 μg/mL ascorbate and 50 µg/ml Cycloheximide for 5 h. Conditioned media were harvested and assessed by collagen IV ELISA Kit, as described in the methods. Relative secreted collagen IV (mean ± SD) was shown and experiments were repeated for three times. Statistical analysis was performed using two-tailed, paired t.test (mean ± SD, *n.s.* not significant).

As accumulation of these vesicular profiles occurred very rapidly within a few hours in peri-Golgi area, we reasoned that these vesicles could have been derived from the Golgi itself, but unable to fuse back with the Golgi, due to lack of the essential Golgi *t*SNARE, Syntaxin5 by Retro-2 treatment. Alternatively, a portion of these vesicles could be COPII vesicles from the ER, but unable to fuse with the Golgi. To help understand the nature of these vesicular profiles, we measured diameter of a large number of these accumulated vesicles and found that they display an average diameter of 57 ± 7.4 nm with a median diameter of 55 nm (n = 213) (Fig. 2A). This measured diameter closely matched the observed size of uncoated COPI-vesicles, which had been shown to be ranging from ~ 60 nm in in vivo studies to 45 ± 6 nm in an in vitro study^28–30^, suggesting that COPI-vesicles may account for a significant fraction of these accumulated vesicular profiles, although we cannot rule out the possibility that a certain percentage of these vesicles may be COPII vesicles, arriving from the ER.

### Anterograde secretion of cargo proteins remains unaffected by Retro-2 treatment

In order to study how Retro-2 treatment and resulting vesicle accumulation may influence basic functions of the Golgi apparatus, we tested cargo secretion using ss-HRP and collagen IV^23^. The results showed that secretion of both small cargo, such as ss-HRP and large cargo, collagen IV, are largely unaffected in the presence of Retro-2 (Fig. 2B–D). These results are consistent with a prevalent view in the field that COPI vesicles are mainly responsible for retrograde transport of Golgi resident glycosyltransferases, whereas cisternal maturation or rim progression may account for anterograde secretion of cargo proteins^31–33^. Taken together, these results indicate that Retro-2 significantly alters Golgi structure to a greater extent than previously anticipated.

### Retro-2 treatment alters Golgi morphology in GRASP65/55-double depleted cells, as examined by structured illumination microscopy

We then considered the possibility that Retro-2 may be exploited to investigate whether Syntaxin5 is required for Golgi structure maintenance in interphase cells. We had previously reported that the two Syntaxin5-binding Golgi stacking proteins, GM130 and Golgin45, can entirely substitute for GRASP65/55 (when overexpressed) to maintain functionally and morphologically normal Golgi stacks in interphase cells^21^. In the previous study, we consistently observed that Golgi cisternae appeared to be significantly dilated, but not unstacked, in cells depleted of GRASP65/55^21^. Furthermore, we also demonstrated that Golgin45-Syntaxin5 interaction contributes to structural integrity of the Golgi stack in interphase cells^24^.

Therefore, we postulated that acute displacement of Syntaxin5 via Retro-2 treatment could be used as a type of ‘knock-sideway’ strategy to investigate the hypothesis that interaction between Syntaxin5 and the two Syntaxin5-binding Golgin tethers may contribute a cisternal adhesion mechanism to restore morphologically and functionally normal Golgi stacking in GRASPs-depleted cells.

Since significant Golgi unstacking occurs only during mitotic Golgi disassembly^9,34–37^ or in cells treated with Golgi-disturbing agents, such as Brefeldin A^3,4^, this experimental approach offers a unique opportunity to test whether Golgin-Syntaxin5 interaction may assist or support Golgi stack maintenance during interphase.

**Figure 3:**
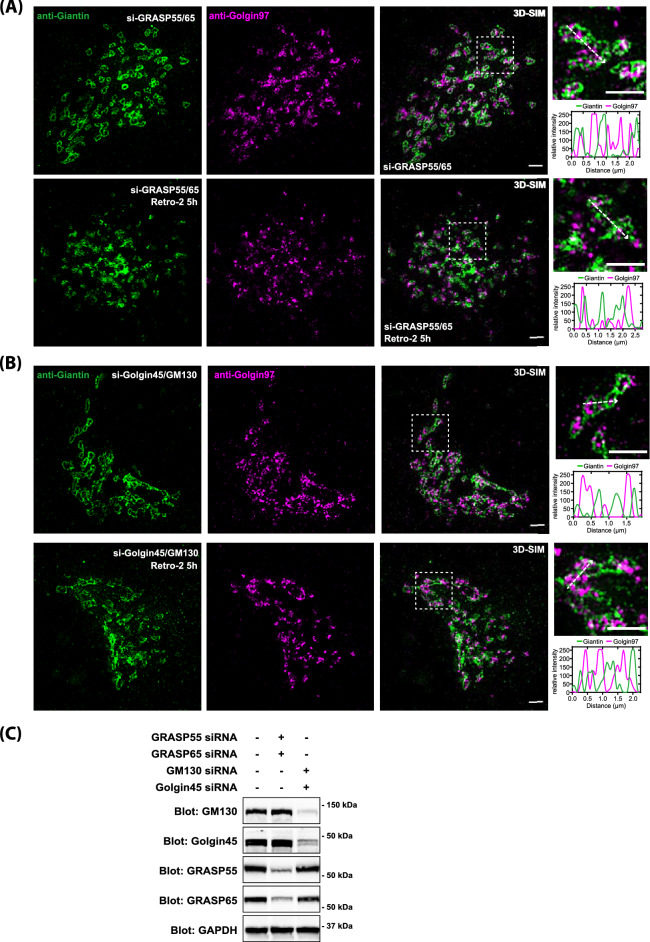
Structured Illumination microscopy data suggest a significant morphological/structural alteration of the Golgi in GRASP65/55-double depleted cells upon Retro-2 treatment. (**A**) 3D-SIM images of the Golgi stained with anti-Giantin and anti-Golgin97 antibodies in GRASP65/55-depleted cells (top panel) and in GRASP65/55-depleted cells, treated with Retro-2 for 5 h (bottom panel). (**B**) 3D-SIM images of the Golgi stained with anti-Giantin and anti-Golgin97 antibodies in GM130/Golgin45-depleted cells (top panel) and in GM130/Golgin45-depleted cells, treated with Retro-2 for 5 h (bottom panel). (scale bar 2 μm). (**C**) Immunoblot analysis of knockdown efficiency of indicated siRNAs used in 3D-SIM experiments.

Thus, HeLa cells were treated with GRASP65/55 siRNAs (Fig. 3A) or GM130/Golgin45 siRNAs (Fig. 3B) for 48 h, followed by Retro-2 treatment for 5 h at 37 °C (Fig. 3C for knockdown efficiency). These cells were then fixed and stained with anti-Giantin and anti-Golgin97 antibodies to examine any change in overall Golgi organization by super-resolution SIM microscopy. Prior to Retro-2 treatment, we observed that Giantin staining always showed distinct peripheral staining pattern with hallow space. However, upon Retro-2 treatment, this distinct feature was largely lost in GRASP65/55-double depleted cells (Fig. 3A; lower panel; as indicated by line analysis at the insets), but not in control or GM130/Golgin45-double depleted cells (Fig. 3B,C for knockdown efficiency).

Taken together, these results suggested that Retro-2 treatment induce greater disruption of Golgi organization in GRASP-depleted cells than in GM130/Golgin45-depleted cells. These changes were quite subtle and difficult to quantify by co-localization analysis, which then prompted us to use EM for more detailed structural analysis.

### Retro-2 induces rapid and complete Golgi unstacking in GRASP65/55-depleted cells within 5 h

**Figure 4:**
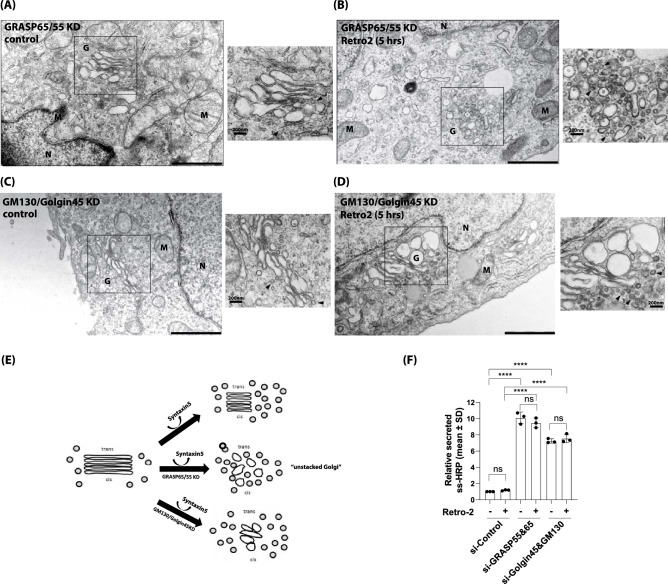
Acute removal of Syntaxin5 from the Golgi by Retro-2 induces Golgi unstacking in GRASP65/55-depleted cells. (**A**,**B**) Representative EM photos showing that Retro-2 treatment induces Golgi unstacking in GRASP65/55-depleted cells. Insets show a magnified view of the boxed area. peri-Golgi COPI vesicles are marked by black arrow heads; (**C**,**D**) representative EM photos showing that Retro-2 treatment does not lead to Golgi unstacking in GM130/Golgin45-depleted cells. Insets show a magnified view of the boxed area; (**E**) illustration summarizing the EM data from Retro-2 treatment experiments using control cells (Fig. 1B), GRASP65/55-depleted cells (**A**,**B**) and GM130/Golgin45-depleted cells (**C**,**D**). Note that acute displacement of Syntaxin5 from the Golgi leads to Golgi unstacking only in GRASP65/55-depleted cells. M = mitochondria, N = nucleus, G = Golgi; (scale bar 1 μm). (**F**) HeLa cells were transfected with GRASP55/65 or GM130/Golgin-45 siRNAs for 48 h and then transfected with ss-HRP overnight. Then the cells were changed with fresh medium and treated with DMSO or Retro-2 for 5 h. The activity of secreted HRP was estimated measured by TMB ELISA, as described in the methods. Relative secreted ss-HRP (mean ± SD) was shown and experiments were repeated for three times. Statistical analysis was performed using two-way ANOVA with a Tukey’s post-hoc test for multiple comparisons (mean ± SD, *n.s.* not significant; ****p < 0.0001).

In order to further study this structural alteration at the ultra-structural level, the cells were then processed for EM study. Strikingly, upon examination by EM, we found that the Golgi was completely unstacked by Retro-2 treatment in GRASP65/55 double-depleted cells, but not in control or in GM130/Golgin45 double-depleted cells (Fig. 4A–E). While Golgi cisterna were still well stacked, they were highly dilated in both GRASP-depleted and Golgin-depleted HeLa cells, which is consistent with our previous observation^21^. These results suggested that Syntaxin5 may contribute to Golgi stack maintenance in GRASP65/55-depleted HeLa cells during interphase. Due to highly heterogenous morphologies of the unstacked Golgi, it was difficult to quantitatively summarize these results, but we found that over 90% of the cells showed similar cisternal membrane de-adhesion in GRASP65/55-depleted cells upon Retro-2 treatment within 5 h. This was not due to disruption of the secretory pathway through the Golgi. Although GRASP double KD and Golgin double KD led to greatly increased ss-HRP secretion, consistent with previous work by ourselves and Wang’s group^21,25^, there were no statistically significant changes in the ss-HRP secretion for GRASP double KD or GM130/Golgin45 double KD cells, treated with DMSO or Retro-2 (Fig. 4F).

### Retro-2-induced Golgi unstacking in GRASP65/55-depleted cells is a time-dependent process

**Figure 5:**
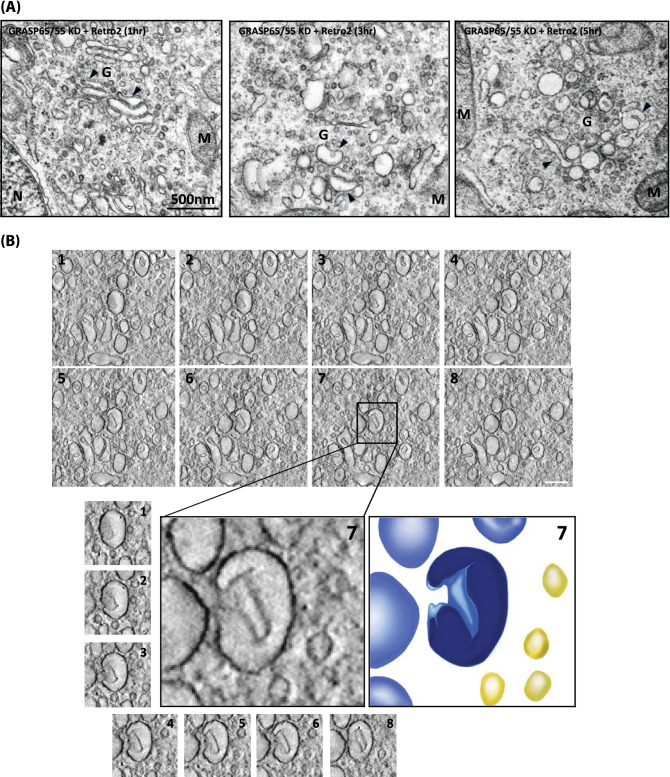
Retro-2-induced Golgi unstacking in GRASP65/55-depleted HeLa cells occurs in a time-dependent manner. (**A**) Representative EM micrographs showing that Golgi unstacking by Retro-2 treatment in GRASP65/55-depleted cells is a time-dependent process. These images show morphological and structural changes of the Golgi in GRASP65/55-depleted HeLa cells after Retro-2 treatment for 1, 3, 5 h, respectively. Black arrow heads indicate Golgi cisternal membranes during Retro-2-induced Golgi unstacking (scale bar 500 nm). (**B**) Sequential slice views from the tomogram showing more detailed structures of the ‘cup’-shaped, unstacked cisternal membranes. Below are the magnified views and segmentation of rendition from one of the unstacked cisternae (light blue; yellow indicates vesicular profiles in peri-Golgi area). (scale bar 200 nm). See movie#S1 for more information.

To more thoroughly characterize this rapid Golgi unstacking, we performed a time-course study using Retro-2 and EM, and found that the alteration of Golgi structure by Retro-2 is a time-dependent process. During the first hour of Retro-2 treatment, we observed only negligible changes in cisternal adhesion (Fig. 5A; left panel). However, there was noticeable cisternal membrane de-adhesion at 3 h of Retro-2 treatment (Fig. 5A; middle panel), which eventually led to almost complete Golgi unstacking at 5 h (Fig. 5A; right panel). Unlike Golgi disassembly during mitosis, Golgi cisternal membranes did not appear to undergo complete vesiculation in Retro-2-treated cells in this time frame and remained as relatively large membranous structures (see Golgi cisternal membranes, indicated by black arrowheads). Most of these membranous structures appeared to be invaginated with hallow space, although a few of them looked like large tubular structures.

In order to further characterize these novel membranous structures, we performed EM tomography of the Golgi in cells depleted of GRASP65/55 and treated with Retro-2 for 5 h. The resulting EM tomographs revealed that the unstacked Golgi membranes were cup-shaped, invaginated membrane structures, typically ranging in 100–250 nm diameter, as shown in EM tomograph tilt series (Fig. 5B; also see the movie#S1 and colored rendition of a cup-shaped membrane structure below).

Although it was difficult to further characterize these unstacked Golgi cisternae in the present study, the unstacked Golgi cisterna in our experiments appears to be strikingly similar to rapidly disrupted Golgi membranes in SARS-CoV-2-infected cells^27^, raising an interesting possibility that SARS-CoV-2 may have an ability to either directly or indirectly disrupt GRASP-mediated cisternal adhesion and alter the intracellular trafficking of certain SNARE proteins for efficient viral replication, which requires future study. In support of this hypothesis, SARS-CoV-2 spike protein has already been shown to directly bind and hijack COPI and COPII for progeny biogenesis^38–40^.

Taken together, our new results indicate that Syntaxin5 may assist Golgin-mediated Golgi structure maintenance during interphase. These results do not unequivocally prove that GM130/Golgin45-Syntaxin5 interaction provides the fundamental mechanism for cisternal adhesion during post-mitotic Golgi reassembly. However, these results do suggest that this unique Golgin-SNARE interaction may contribute to Golgi structure maintenance during interphase by inhibiting intercisternal fusion among adjacent Golgi cisterna and by simultaneously providing additional support for cisternal adhesion.

**Figure 6:**
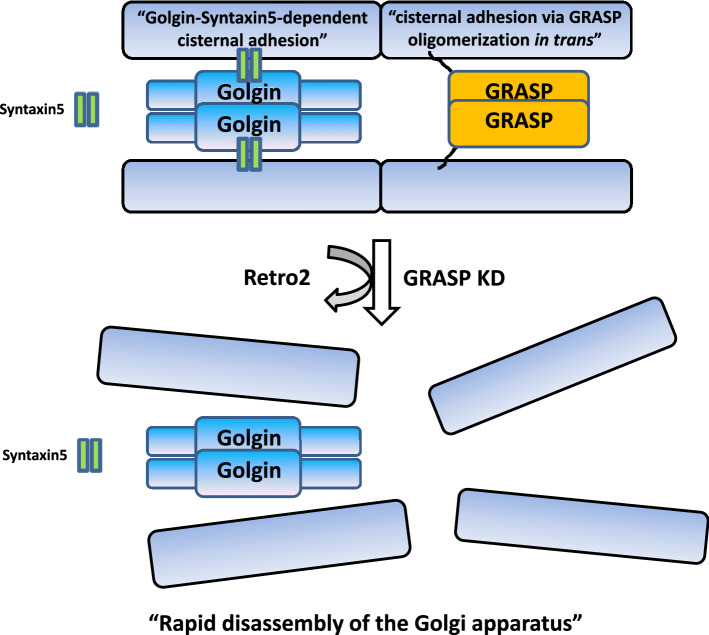
A model depicting how Golgin-Syntaxin5 interaction may assist or support cisternal adhesion during interphase. We suggest that GRASP-dependent cisternal adhesion and direct interaction between Syntaxin5 and Syntaxin5-binding Golgin tethers likely support cisternal adhesion in incremental or additive manner. In GRASP-depleted cells, Retro-2 treatment leads to complete disassembly of the Golgi within a few hours, because of rapid displacement of Syntaxin5 from the Golgi. It is noteworthy that this unique Golgin-tSNARE interaction may not only assist cisternal adhesion during interphase, but also influence the frequency of inter-cisternal fusion among closely apposed Golgi cisterna or lateral linking of stacks for Golgi ribbon formation.

Thus, we propose that direct interaction between Syntaxin5 and Golgin45/GM130 (or any other Syntaxin5-binding Golgin tethers) may assist Golgi structure maintenance during interphase, as illustrated in the model (Fig. 6). For a mechanistic point of view, this would be quite distinct from GRASP65/55-dependent cisternal adhesion, where GRASP65/55 contribute to cisternal stacking via their PDZ-domain-mediated homo-oligomerization in trans between two apposed cisternae^9,10,34,41,42^. Instead, Golgin-SNARE interaction may provide a weak reversible binding, facilitating dynamic membrane adhesion/de-adhesion for regulation of cargo transport through the Golgi and basic Golgi structure maintenance. It would be especially intriguing to investigate in the future how this proposed role may intertwine with recently reported Syntaxin 5 phosphorylation under proteostatic stress^43,44^.

Overall, these new results implicate Syntaxin5 in Golgi structure maintenance and regulation during interphase, which may have been overlooked thus far. As Syntaxin5 is localized throughout the Golgi stacks, we suggest that the Golgi *t*SNARE may be optimally positioned to perform such a structural role.

## Methods

### Reagents and antibodies

All common reagents were purchased from Sigma-Aldrich, unless otherwise mentioned. The following antibodies were used : anti-Giantin (ab174655, Abcam), anti-Golgin97 (A21270, Thermo), anti-GM130 (610822, BD bioscience), anti-GRASP65 (MA5-25148, Thermo), anti-GRASP55 (10598-1-AP, Proteintech), anti-Golgin45 (PA530714, Thermo), anti-Syntaxin 5 (110053, Synaptic Systems), anti-GAPDH (KC-5G5, Kangchen Bio-tech). Retro-2 was obtained from Sigma-Aldrich. Anti-Rabbit Alexa Fluor 488 (A21441), Alexa Fluor 568 (A10042), Alexa Fluor 647 (A21245) and anti-Mouse Alexa Fluor 488 (A21200), Alexa Fluor 568 (A10037), Alexa Fluor 647 (A21236) for immunofluorescence were obtained from ThermoFisher. All siRNA oligos were purchased from Shanghai GenePharma, China and the target sequences were as following: human Golgin45 (GGGAACAGTTTCGTCAAGA), human GRASP55 (GGCATTGGATATGGTTATT), human GM130 (GGACAATGCTGCTACTCTACAACCA), human GRASP65 (CCTGAAGGCACTACTGAAAGCCAAT). The sequence of the non-targeting control siRNA was UUCUCCGAACGUGUCACGU.

### Cell culture and treatments

HeLa (ATCC, CCL-2) and COS7 (Stem Cell Bank, Chinese Academy of Sciences) cells were grown in DMEM supplemented with 10% FBS (Thermo) at 37 °C. HeLa cells were authenticated by STR profiling. The authentication of COS7 is provided by Stem Cell Bank, Chinese Academy of Sciences. All cell lines were routinely tested for the mycoplasma contamination and were negative. Transfection of DNA constructs and siRNAs was performed using Lipofectamine 2000 and RNAiMAX (ThermoFisher), respectively, according to the manufacturer’s instructions.

### Immunofluorescence staining

Cells grown on glass coverslips (72,230–01, Electron Microscopy Sciences) in 24-well plates were fixed for 10 min with 4% paraformaldehyde (PFA), permeabilized in permeabilization Buffer (0.3% Igepal CA-630, 0.05% Triton-X 100, 0.1% IgG-free BSA in PBS) for 5 min, and blocked in blocking buffer (0.05% Igepal CA-630, 0.05% Triton-X 100, 5% normal goat serum in PBS) for 60 min. Primary and secondary antibodies were applied in blocking buffer for 1 h. The nucleus was stained with Hoechst-33342 (sc-200908, Santa Cruz Biotechnology). Cells were washed three times with wash buffer (0.05% Igepal CA-630, 0.05% Triton-X 100, 0.2% IgG-free BSA in PBS) and twice with PBS. Coverslips were mounted using ProLong Gold Antifade Reagent (ThermoFisher). Dip coverslip in diH_2_O before mounting to prevent salt contamination. Images were acquired with a Zeiss LSM880 confocal microscope using a 63 × Apochromat oil-immersion objective. 3D-structured illumination microscopy (SIM) imaging was acquired using Nikon N-SIM microscope.

### Immunoblotting

For immunoblotting, proteins were separated by SDS-PAGE (Genscript) and transferred onto nitrocellulose membranes (Amersham). Membranes were blocked with 3% bovine serum albumin (BSA) and then probed with specific primary antibodies and then with peroxidase-conjugated secondary antibodies (Jackson ImmunoResearch). The bands were visualized with chemiluminescence (Clarity Western ECL Substrate, Bio-Rad) and imaged by a ChemiDoc Touch imaging system (Bio-Rad). Representative blots are shown from several experiments.

### Sample preparation and image acquisition for Electron microscopy/tomography

The cells were fixed in 2.5% glutaraldehyde in 0.1 M sodium cacodylate buffer pH7.4 for 1 h. They were then rinsed in 0.1 M sodium cacodylate buffer, scraped and pelleted in 2% agar. Samples were trimmed and post-fixed in 1% osmium tetroxide for 1 h, en bloc stained in 2% uranyl acetate in maleate buffer pH5.2 for a further hour, rinsed then dehydrated in an ethanol series and infiltrated with resin (Embed812, Electron Microscopy Science) and cured overnight at 60 C°. Hardened blocks were cut using a Leica UC7 Ultramicrotome, 60 nm sections were collected onto formvar/carbon coated nickel grids and stained using 2% uranyl acetate and lead citrate. These were viewed FEI Tecnai Biotwin TEM at 80 kV. Images were taken using Morada CCD and iTEM (Olympus) software typically at 26,000 × magnification. For electron tomography, 250 nm sections were collected on formvar/carbon copper grids, 10 nm gold particles added on both sides of the grids (Utrect UMC). A tomography tilt series was acquired using SerialEM software on an FEI Tecnai TF20 FEG TEM at 200 kV. tomograms were reconstructed using IMOD software (University of Colorado, Boulder, CO).

### ss-HRP and collagen IV secretion assays

After overnight transfection with ss-HRP plasmid, the cells were changed with fresh medium and treated with DMSO or Retro-2 for 5 h. Extracellular media were harvested and HRP activity was measured using 1-Step Ultra TMB-ELISA (ThermoFisher), according to the manufacturer’s instructions. For collagen IV secretion, COS7 cells pre-treated with DMSO or Retro-2 for 2 h before folding block (40 °C, 3 h) without ascorbate and transport pulse condition, was later induced by shifting cells to 32 °C in the presence of 100 mg/mL ascorbate and 50 µg/ml Cycloheximide for 5 h. Extracellular media were harvested and assessed by collagen IV ELISA Kit (FineTest, EH2867), according to the manufacturer’s protocols.

### Image processing and data presentation

Line intensity of 3D-SIM images were analyzed by Fiji software. Results are displayed as mean of results from each experiment or dataset, as indicated in figure legends. Analyses were performed with GraphPad Prism 9.0 software.

## Supplementary Information

Supplementary Video 1.

Supplementary Figure 1.

## Data Availability

The datasets used and/or analyzed during the current study is available from the corresponding author on reasonable request.
