# Supplementary material for: Retro-2 alters Golgi structure
Source: Sci Rep. 2022 Sep 2;12:14975. doi: 10.1038/s41598-022-19415-x (PMC9438350; doi:10.1038/s41598-022-19415-x)

Supplementary Material

Supplementary Figure 1. unprocessed images for all Western blot data

Supplementary Movie#1. Movie file showing the EM tomogram tilt series in Figure 5B

Supplementary Figure 1
Unprocessed images of all blots

Unprocessed western blots in main figures. Some blots were cut into several pieces and incubated with different antibodies.

Unprocessed blots of Figure 3C

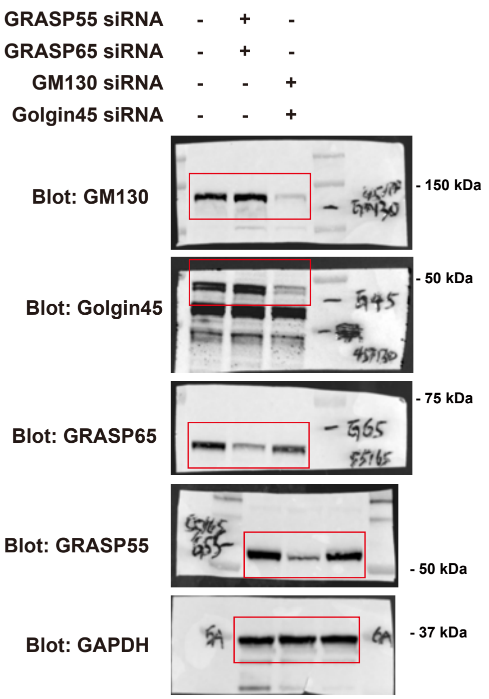

Supplement: Supplementary file 2 — Supplementary Figure 1. [file 41598_2022_19415_MOESM2_ESM.pdf]
